# The Symptomatic Significance of Apparent Paralysis of the Hind-Quarters in the Horse

**Published:** 1880-01

**Authors:** Charles A. Meyer

**Affiliations:** Consulting Surgeon to the Hospital Department of the Columbia Veterinary College


THE ARCHIVES

OF

COMPARATIVE MEDICINE AND SURGERY.

Vol. I. JANUARY, 1880. No. 1.

### Original Articles, Selections, and Translations.

### Art. I.—THE SYMPTOMATIC SIGNIFICANCE OF APPARENT PARALYSIS OF THE HIND-QUARTERS IN THE HORSE.

By CHARLES A. MEYER, D. V. S.,

Consulting Surgeon to the Hospital Department of the Columbia Veterinary College.

THERE is, probably, no single symptom in the horse which has led to more vague generalization as to diagnosis, and more crude measures as to therapeutics, than the real or apparent paresis of the hind-legs. The difficulties in the proper interpretation of this symptom are certainly not insurmountable, and yet we find the most varied affections in which it appears, either as a symptom or as a complication, thrown together under the common term "cerebro-spinal meningitis" or, perhaps, of "spinitis." When the reader will bear in mind that even veterinarians of "great" experience have diagnosticated as cerebro-spinal meningitis what, on the post-mortem, revealed itself as a rupture of the *quadratus lumborum* muscle, or a wire which had been run in the foot (!), or a typical case of suppurative nephritis, they will agree with the writer that, *as frequently used*, this term is but a cloak for ignorance.

Some of those text-books which are, to too great an extent, the sole reliance of sundry regular and irregular practitioners, are in a measure responsible for the current notion, that wherever there is dragging of the posterior extremities, disinclination to walk, and inability to rise without assistance, the horse suffering from these symptoms must have spinal meningitis. But what shall be said when a veterinarian, once a teacher in a school of comparative medicine, actually instructs his class to the effect that you may have anaemia or hyperaemia of the cord in spinal meningitis, and that in the former condition the conjunctiva is pale, in the latter injected? What ideas as to spinal pathology and physiology must be flitting through the mind of him who, after making such statements in a formal lecture, a few weeks after diagnosticated as spinal meningitis a case of typical haemoglobinuria?

These few statements seem to the author to justify a brief exposition of the true significance of that symptom which is most frequently considered to establish the existence of spinal or cerebro-spinal meningitis, however erroneously.

In doing so the writer is well aware that he is but treading in the beaten path in which the classical British and Continental authorities have long preceded him. His sole hope is, that in the present merely provisional communication the student of veterinary medicine, as well as the practitioner to whom the European periodicals are not accessible, may find the facts, scattered in various treatises, conveniently condensed. In a future article he proposes to discuss the subject more exhaustively and from other points of view.

A few cases from among those which have fallen under the author's observation may serve to illustrate the statement made, that paralysis, *per se*, is a very deceptive symptom, and may exist with affections of the most widely different character. They also furnish the text to some remarks of a general nature, which conclude the paper.

*Case I.*—A black gelding—age, 6 years; in splendid condition; weight, 1400 pounds—was brought to the infirmary in an ambulance. The history of the case was, that, having exhibited no untoward symptoms in the morning, and having fed as usual, it was harnessed to a buggy, and after being driven about a thousand feet, dropped suddenly. On pricking the hind-legs with a pin, no reaction took place, and the animal was found unable to rise to its feet, even with assistance, at the infirmary. When persuaded by the lash, it made endeavors to rise, sitting up on its haunches and falling over in the attempt to rise further. The animal seemed in great pain, and the posterior extremities were very cold. Convulsions finally set in, and the horse died during the same night in protracted agony.

The case was under the charge of the then house-surgeon of the infirmary, who pronounced it spinal meningitis, and proceeded to treat it as such. It was observed that the administration of any remedies whatever aggravated the animal's distress.

Post-mortem eight hours after death. Brain and spinal cord were found to be perfectly healthy, and the meninges showed nothing beyond an anaemic condition in the lumbar region. A microscopic examination of the cord, from the origin of the second lumbar nerve, failed to reveal the slightest abnormality. The left kidney was pale, its capsule easily came off, and with the right one gave off a peculiar odor. The right kidney was enlarged and softened, all its tissues, on section, exhibited a darkish-green tinge, its centre near the papillae was completely broken down, and numerous small abscesses, filled with a yellow or yellowish-green pus, and ranging in size from that of a millet-seed to that of a cherry, were found throughout the remaining cortical and medullary substance. The other organs were healthy; there was a small amount of fœtid and discolored urine in the bladder. The muscles of the lumbar region were oedematous, and on removal from the body were noticed to emit strongly the same peculiar odor noticed in the case of the kidneys. I should characterize this odor as being neither decidedly urinous nor gangrenous, but combining the two with an altogether indescribable sweet, mawkish smell.

The anatomico-pathological diagnosis was acute suppurative nephritis.

It is very much to be regretted that those in charge of the case saw no occasion for examining the urine. Such an examination would have immmediately directed attention to the seat of the malady. But even as it was the history of the case would have forbidden the making of the diagnosis mentioned as given during life. The sudden onset of the paraplegia alone would exclude spinal meningitis, a disease in which paralysis is as a rule secondary to hyperæsthesia and hyperkinesis. Sudden paralysis of the hind-legs points to either hemorrhage in the spinal cord * or around it, in the sac of the arachnoid. Or it may be due to acute myelitis with or without consecutive hemorrhage. Finally, it may

* Trasbot, Recueil de méd vétèr, Mars, 1870. Paraplégie aigue, duc à une congestion de la moelle épinière an reflement lombaire chez le cheval.

be a reflex paralysis, and this is the explanation for our case. There are important physiological connections between the organs of the genito-urinary system and the spinal cord. The researches of, particularly, the French physiologists have demonstrated that irritation of the kidneys or renal nerves may produce sudden paraplegia in dogs, (Comhaire, Brown-Sequard,) and Lewisson's more thorough researches have clearly shown that the paraplegia appears, continues and disappears *pari passu* with the irritation of the kidneys. Case I. is, therefore, a typical example of *Reflex paralysis as a symptom of kidney disease.*

*Case II.*—A large, heavy draught mare in good condition (apparently) suddenly dropped while at work, and with difficulty could be brought back to its stall. The bladder was found distended with urine, which, on being drawn off with the catheter, was found to have a strong ammoniacal odor, and a coffee-color with a reddish tinge; it was also of a sticky consistency. The veterinarian called in to see this case, and who obliged me with communicating his observations, diagnosticated and treated this case as one of "spinal meningitis." I was called in to see the case, and found the following: Profuse perspiration, loss of motor power in the hind legs, increased frequency of respiration, pulse accelerated, membranes injected, fascicular twitches of the gluteal muscles, inability to void the urine or fæces.

Even on the fifth day she ate her feed as usual, then her owner had her destroyed, on his own responsibility, and sent the kidneys to the writer, as requested. Beyond a marked hyperaemia, these organs showed nothing unusual.

That this was a case of Haemoglobinuria (Bollinger) sometimes termed Azoturia, there cannot be any doubt. Bollinger here considers the paraplegia as due to the collateral oedema of the lumbar muscles, and perhaps of the lumbar enlargement of the cord. Whether we accept this explanation or not, it is established, and this was our object in introducing the case, that paralysis of the hind-legs may be a symptom of that blood disease, analogous to the haemoglobinuria of cattle, which Bollinger * has aptly termed "toxaemic haemoglobinuria."

*Case III.*—A six-year old gelding was suddenly seized with pain in the back, and abdominal region (judging by her actions); would frequently lie down and get up, stumbling around and lying down again,

* O. Bollinger. Uber Haemoglobinurie (Schwarze, Harnwinde, Windrehe) beim Pferde. Dutsche Zeitschrift für Thiermedizin und vergleichende Pathologie. Band III.

all the while in evident distress. Extreme hyperaesthesea on touch, and copious perspiration. Refused food, but showed a craving for dirt, chewing away at all the wood-work within reach, and biting the mortar from the walls. The distress continued, the symptoms became more alarming, tetaniform convulsions came on, and on the third day the animal died, having become paraplegic. The owner, suspecting strychina poisoning by a discharged groom, desired a post-mortem, which, on being held, gave the following results: Stomach and small intestines moderately injected, probably from the irritation caused by the foreign articles swallowed. Liver, spleen, lungs, heart, kidneys, and bladder healthy. Brain normal, medulla oblongata ditto. The spinal cord intensely injected, both in its substance and its meninges, the pia as well as the dura were purplish, the peri-dural tissue reddened, and the cerebro-spinal fluid tinged with blood. This condition was limited to that part of the cord between the origin of the second dorsal nerve and the cauda equina. From the level of the fourth to the seventh dorsal vertebra the cord was softened, its tissue almost in a condition of fluidification, and of a slight chocolate tinge in the gray substance, though white in the columns of white matter. Anatomico-pathological diagnosis: Acute myelitis of the dorsal portion, with congestion of the entire dorso-lumbal cord and its meninges. Microscopic examination of the softened portion revealed granule cells, broken-down myelin sheaths and detritus. Subsequent inquiry revealed the fact that certain wet bandages applied to the hind-legs of this horse by the owner, the day previous to its admission in the infirmary, had been permitted to become cold on the animal, on a November day, so that evidently we here had a case of myelitis from cold.

This case illustrates that paraplegia may be a symptom of organic spinal disease without complicating meningitis.

An old, worn-out mare, very weak in her hind-legs, which was purchased for the dissecting table, was found to have suddenly become paraplegic. On account of its feeble condition this symptom was overlooked, and being destroyed, the mare was placed on the dissecting table. Quite accidentally, a tumor of a peculiar shape, and proceeding from the *dura mater* covering the tentorial protuberance, about as thick as a walnut, but twice as long, was found lodged in an excavation of the compressed cerebral substance, just to the left of the median line. There was also considerable oedema of the brain and cervical cord. The tumor being unfortunately lost, no microscopic examination could be made. On macerating the skull, two large spicula were found at the point of the parietal protuberance corresponding to the situation of the tumor.

This may be regarded as paraparesis matured into paraplegia, whose primary cause was a *tumor of the cerebral membranes.* It is well known that crossed hemiplegia, the classical result of unilateral disease of the brain in man (and the monkey, according to

Ferrier's experiments and certain pathological experiences,) is rare, if ever present in the horse, hemiparesis of the same side, or paraparesis, are the ordinary result.

It would be nothing less than insulting to the intelligence of our readers to go over a host of other affections in which either paralysis, convulsions, or distortions of the hind-quarters occur, and which have not the remotest relation to that universal refuge of the bewildered, "spinal meningitis." The cases given will, however, serve to show the varied character of the disease processes which may present these symptoms. But we cannot refrain from saying that we have actually seen cases of vertigo (epileptic), tetanus, mania, diabetes, lumbar sprain, purpura, rheumatism, and influenza designated as spinal meningitis, on no stronger grounds than the presence of paraplegia. It may be but little flattering to our national pride to admit that the average veterinarian on this side of the Atlantic is more in the habit of resorting to a haphazard diagnosis than of carefully and logically analyzing the relation between lesion and symptoms! The following propositions may serve as a basis for a more extended discussion of this suggestive topic:

1. Paralysis is a symptom only, never a disease itself.

2. Paralysis in the hind-members of a horse may be real or apparent.

3. The intricate character of the locomotor mechanism in the horse renders it possible for purely mechanical disturbances, rupture of muscles, etc., to simulate paresis or paralysis to a superficial observer. And if the animal suffering from such disorder present, in addition, a slight rise in temperature, the diagnosis of spinal meningitis appears confirmed to such. It is unnecessary to enter into the details of differential diagnosis here; no one familiar with the action of the muscles, or possessed of the faintest idea of pathology, or who had even empirically seen a considerable number of diseased animals, would fall into such a gross error. This disposes of "apparent" paralysis of mechanical origin.

4. True paralysis and paresis may be due to disturbances of the brain cord and peripheral nerves.

5. In the shape of sudden hemiparesis or paraparesis, it may develop with cerebral or meningeal hemorrhage, or cerebral traumatism.

6. In the form of rapidily produced though not instantaneous paraplegia, it is the essential symptom of acute myelitis. Conscious sensibility is here entirely suspended, a prick of a pin will however cause the most violent spasms in the hind-legs, owing to the increased *reflex* excitability. The test of pricking the parts to see whether sensation remains, is therefore fallacious.

7. Paraparesis gradual in its development, and subject to considerable change in the history of a given case, may occur with Hyperaemia of the cord. This explains the staggering of horses noticed when suddenly brought from a warm to a cold atmosphere, a symptom that may be evanescent or lead to graver developments.

8. It may be a reflex paralysis, as with renal disease, and possibly cases may yet be found in which irritation of more distant parts, as in man, may bring about this symptom.

9. A failure in the proper blood supply, or in its composition, may induce a similar result. Embolus or Thrombus of the posterior aorta, Uraemia and Diabetes, act in this way. The paralysis produced by embolus or thrombus usually occurs in two ways; first, by defective nutrition of the cord; second, by defective nutrition of the muscles.

10. In spinal meningitis (a far rarer affection than is usually supposed) whether idiopathic or complicating epidemic febrile affections, paraplegia may also occur; but it is a secondary symptom, and preceded by a stage of excitement with hyperaesthesia and hyperkinesis. The paralysis usually does not involve both hind limbs in the same degree, or the different muscles of the same limb equally.

With these few suggestions, which are intended less for the experienced practitioner than for the student, and each of which alone, to have justice done it, would require a separate essay, I close this communication, trusting to resume the discussion of the subject at no distant time.

